# Comparative evaluation of network features for the prediction of breast cancer metastasis

**DOI:** 10.1186/s12920-020-0676-3

**Published:** 2020-04-03

**Authors:** Nahim Adnan, Zhijie Liu, Tim H.M. Huang, Jianhua Ruan

**Affiliations:** 1 Department of Computer Science, University of Texas at San Antonio, One UTSA Circle, San Antonio, TX 78249 USA; 2 Department of Molecular Medicine, University of Texas Health Science Center at San Antonio, 7703 Floyd Curl Drive, San Antonio, TX 78230 USA

**Keywords:** Breast cancer metastasis, Metastasis prediction, Network features, Gene expression analysis

## Abstract

**Background:**

Discovering a highly accurate and robust gene signature for the prediction of breast cancer metastasis from gene expression profiling of primary tumors is one of the most challenging tasks to reduce the number of deaths in women. Due to the limited success of gene-based features in achieving satisfactory prediction accuracy, many methodologies have been proposed in recent years to develop network-based features by integrating network information with gene expression. However, evaluation results are inconsistent to confirm the effectiveness of network-based features, because of many confounding factors involved in classification model learning process, such as data normalization, dimension reduction, and feature selection. An unbiased comparative evaluation is essential for uncovering the strength of network-based features.

**Methods:**

In this study, we compared several types of network-based features obtained using different mathematical operators (Mean, Maximum, Minimum, Median, Variance) on geneset (i.e., a gene and its’ neighbors in the network) in protein-protein interaction network and gene co-expression network for their ability in predicting breast cancer metastasis using gene expression data from more than 10 patient cohorts.

**Results:**

While network-based features are usually statistically more significant than gene-based feature, a consistent improvement of prediction performance using network-based features requires a substantial number of patients in the dataset. In contrary to many previous reports, no evidence was found to support the robustness of network-based features and we argue some of the robustness may be due to the inherent bias associated with node degree in the network. In addition, different types of network features seem to cover different pathways and are complementary to each other. Consequently, an ensemble classifier combining different network features was proposed and was found to significantly outperform classifiers based on gene-based feature or any single type of network-based features.

**Conclusions:**

Network-based features and their combination show promise for improving the prediction of breast cancer metastasis but may require a large amount of training data. Robustness claim of network-based features needs to be re-examined with network node degree and other confounding factors in consideration.

## Background

The most frequently diagnosed disease and the second leading cause of death in western women have been identified as breast cancer [1]. According to the American Cancer Society [2], US women have 12.4% (about 1 in 8) chance of developing breast cancer over the course of their lifetime. About 5% of women have metastatic (i.e., recurrence of cancer) breast cancer at their first diagnosis [3]. Only lymph node status, histology and tumor size of the patients are not sufficient to determine breast cancer metastasis [4]. Due to the availability of gene expression data for primary cancerous tumors, many methods have been developed to predict breast cancer metastasis outcomes over the last decade. The patient being free from recurrence for at least 5 years and relapse occurring within 5 years after the first diagnosis are termed as good and poor outcomes respectively.

Initially, single gene-based prognostic signatures that are highly differential between good and poor outcomes were proposed [1, 5–7]. Inconsistencies of prognostic signatures were discovered when single gene-based prognostic signatures varied in different studies [8]. There was no homogeneity within the found signatures which complicated the biological relevance of the metastatic outcomes. A lot of genes are correlated with the metastatic outcome which makes it possible to identify multiple single gene-based signatures from the same dataset [8, 9]. Another problem in the gene expression analysis is that the data available is very high dimensional, for example- the number of genes is 10,000 for only hundreds of patients. Many studies tried to pool multiple datasets together to lessen the n >> p problem of the expression data and it provided higher statistical significance of the results [9–11]. Many samples are needed to identify robust gene signature and to overcome the issues of dataset heterogeneity. The number of samples needed to achieve an improvement on classification accuracy is still unexplored and more studies are required to accurately address this concern.

From a biological standpoint, protein works in complexes and the aberrant nature of these complexes can cause cancer. Motivated by this assumption, many methodologies have been proposed integrating network information such as- protein-protein interaction network, co-expression network and cellular pathway information with the gene expression data for better classification accuracy and robust gene signature identification [12–22]. By integrating network with gene expression, those methods tried to find protein complexes or genesets which better distinguished between the good and poor outcome of the metastasis. The aggregated expression of the genes belonging to a geneset was used as the network-based feature in most of the methods. An initial feature selection step was applied to refine the network-based features before creating the final classification model in some of the previously proposed methods [12, 16].

Although many studies claim improved classification accuracy and gene signature stability, there has been insufficient comparative analysis between network-based methods and single gene-based approaches. Two recent studies [23, 24] compared single gene-based approach with the network-based ones and reported that network-based methods do not perform better over the single gene-based approach in terms of classification accuracy and the signature stability. Later, in [21], the authors pointed out that selecting network-based features based on statistical significance hinders the classification performance of the network-based methods. They also argued that only using the average operator to create network-based features does not improve classification accuracy significantly. Other types of operator (i.e., maximum, minimum, median, variance) should also be used in the models.

The current study mainly focused on the differential analysis of the different feature types by comparing network-based features with the single gene-based feature. Amsterdam Classification Evaluation Suite (ACES) [24], a compilation of gene expressions of twelve (12) separate studies, was used for the analysis. The goal of the study was to determine the number of significant features that passed the significance threshold value for separate studies. The impact of the number of samples, classification accuracy of feature types and robustness of gene signature across studies were thoroughly examined. An ensemble classifier CNF (**C**ombining multiple **N**etwork-based **F**eatures) was proposed which provides improved performance over individual network-based features. Moreover, Gene Ontology analysis was done to find the biological interpretation of the top significant genes from separate studies.

## Methods

### Gene expression data

The gene expression dataset used in this analysis is ACES [24] dataset. It combined breast cancer patient samples from 12 different studies together from NCBIs Gene Expression Omnibus. The dataset took the 133A platform into account and removed duplicate samples with the same GEO id in multiple studies. Sample array quality control checking was done, and outlier samples were discarded after RLE (Relative Log Expression) or NUSE (Normalized Unscaled Standard Error Plot) analysis. This yielded a cohort of 1616 patients from 12 studies and all-together the patient expression arrays were normalized using *justRMA* method from *R*. Probe intensities of samples were log-normalized and mean centered. Finally, after discarding missing, null or zero values there were 12,750 gene probes in ACES dataset [24]. The class label for each patient was determined as good or poor outcome based on the recurrence free survival time where the threshold was set to 5 years. The detailed information about the studies and the class distributions are provided in the Table 1.

**Table 1:** Specification of the studies in ACES

Dataset	Geo accession no.	No. of poor	No. of good	Total patient
Desmedt	7390	56	127	183
Hatzis	25066	102	48	150
Ivshina	4922	30	72	102
Loi	6532	24	33	57
Pawitan	1456	33	114	147
Miller	3494	21	68	89
Minn	2603	21	44	65
Schmidt	11121	24	145	169
Symmans	17705	37	187	224
WangY	5327	10	42	52
WangYE	2034	88	169	257
Zhang	12093	9	112	121
ACES		455	1161	1616

### Protein-protein interaction network

The protein-protein interaction network (PPI) was created from the BioGrid (version 3.4.149) interaction database [25]. The interaction network only contained the genes from the ACES dataset where self-edges were discarded which finally produced 180,371 edges for 12,750 nodes (no. of genes).

### Gene co-expression network

A global co-expression network was created for the analysis based on mutual k-nearest neighbors of genes using Pearson correlation coefficients between the genes’ expression in the ACES dataset (i.e., two genes were connected if they were within the top-*k* most co-expressed genes from each other.) The number of neighbors (*k*) was set to 84 so that the network can have a similar number of edges as in the PPI network. This yielded a network containing 161,042 edges for 12,750 nodes (no. of genes) and the degree distribution approximately followed a power-law distribution like the PPI network.

### Feature types

Each gene and its’ neighbors from the network were considered as a geneset. Multiple network-based features were evaluated using different mathematical operators (MAX, MIN, MEDIAN, MEAN, VARIANCE) on the geneset. The specification of the feature types is given in Table 2, where abbreviation “CE” stands for Co-Expression network and “PPI” for Protein-Protein Interaction network. CEEdge and PPIEdge are the two feature types that consider each of the edges of network as features.

**Table 2:** Specification of the feature types

Name	Details
Gene	Using gene expression without integrating any network information.
CENO	A genes’ expression is based on the average expression of its neighbors only.
CEMEAN	The mean of the expression of a gene and its neighbors.
CEMAX	The maximum of the expression of a gene and its neighbors.
CEMIN	The minimum of the expression of a gene and its neighbors.
CEMED	The median of the expression of a gene and its neighbors.
CEVAR	The variance of the expression of a gene and its neighbors.
CEEdge	Each edge is the summation of the expression of its corresponding genes.
PPINO	A genes’ expression is based on the average expression of its neighbors only.
PPIMEAN	The mean of the expression of a gene and its neighbors.
PPIMAX	The maximum of the expression of a gene and its neighbors.
PPIMIN	The minimum of the expression of a gene and its neighbors.
PPIMED	The median of the expression of a gene and its neighbors.
PPIVAR	The variance of the expression of a gene and its neighbors.
PPIEdge	Each edge is the summation of the expression of its corresponding genes.

### Identification of significant features

First, for each feature, its value is calculated for each patient depending on the feature types (see Table 2). Student’s t-test is then used to compare feature values between the good and poor outcome patients in a dataset, and a *p*-value is computed for each feature. Then, we computed False Discovery Rate (FDR) based on Benjamini and Hochberg method [26] and an FDR corrected *p*-value threshold was set to 0.1 for selection of significant features.

### Robustness measure of features

To evaluate the robustness for different feature types across different studies, we selected the top-160 most statistically significant features (ranked by Student’s t-test *p*-value) from each gene- or network-based features (except CEEdge and PPIEdge). The number 160 was chosen so that the expected overlap between the features from two different datasets is 160×160/12750=2 (160 genes chosen from 12,750 genes). For CEEdge and PPIEdge, we pooled the genes associated with the top-ranked edges so that a total of 160 genes were obtained. Then the actual overlap of genes between each pair of datasets was counted, and the geometric mean of the fold change (observed overlap / expected overlap) across the 66 pairs of datasets was calculated as a measure of the feature’s robustness.

### Classification model and performance evaluation

Logistic regression was used as the classification model for evaluating the prediction performance of different feature types. The area under the receiver-operating characteristics curve (AUC) was used as the performance measurement of the classification model due to the class imbalance nature of the data. For evaluation purpose, average AUC of 10 repetitions of 5-fold cross-validation was measured for each feature type in each dataset.

### Classifier with combined network features (CNF)

Based on the classification performance and mutual complementariness of individual network-based features, an ensemble classifier based on combined network-based features, CNF, was proposed. To acquire better confidence in classification, CNF utilizes multiple network-based features together, including MEAN, MAX, MIN, MEDIAN features for both co-expression network and PPI network. A logistic regression model is created for each of the eight network-based feature types. When a test instance is provided, CNF obtains predicted probabilities from the eight logistic regression models for that test instance. The final prediction for that particular test instance is done by averaging the predicted probabilities of those individual classifiers.

## Results and discussion

### Number of significant features

**Fig. 1:**
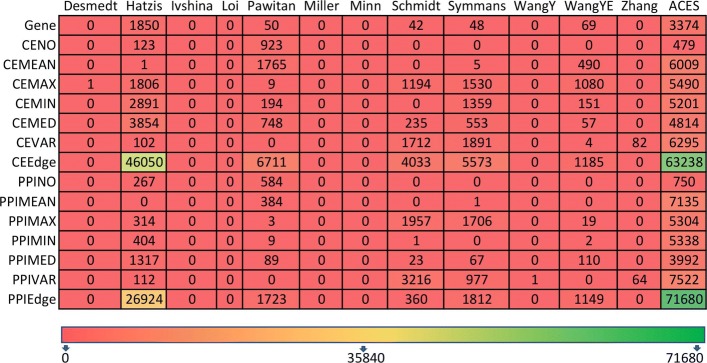
Number of significant features for each feature type in 13 datasets

**Fig. 2:**
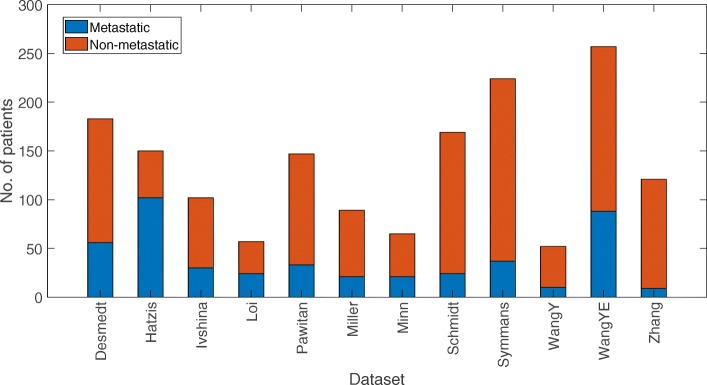
Patient class distribution in 12 studies

Figure 1 shows the number of features passing the FDR corrected *p*-value threshold for each feature type in the 12 cohorts, as well as in the combined *ACES* dataset. First, almost none of the features were able to pass the threshold in “Desmedt”, “Ivshina”, “Loi”, “Miller”, “Minn”, “WangY”, “Zhang” datasets. This indicated that the number of patients and the class distribution have an impact on feature significance. From Fig. 2 it can be observed that “Ivshina”, “Loi”, “Miller”, “Minn”, “WangY”, “Zhang” have the lowest number of patients and very imbalanced class distribution. Although the “Desmedt” dataset has relatively more patients and the class distribution is less skewed, no features passed the FDR corrected *p*-value threshold, indicating that the differential analysis also depends on the nature of the dataset. Note that the total number of CEEdge and PPIEdge features are much larger than other network-based features, hence these feature types have the highest number of significant features.

The result of the *ACES* dataset seems much more stable for each feature type compared to the results of the separate studies. The number of gene features passing the predefined threshold is much higher than separate studies (Fig. 1). Uniform significant number of features for *ACES* dataset indicated that a large number of samples is required for attaining the consistent result. The number of significant features for most network-based feature types (except CENO and PPINO) was much higher than the gene-based feature in *ACES* dataset, suggesting that network neighbors can provide additional discriminating information that would otherwise be too noisy to identify for individual genes. It is also worth noting that network features resulted from multiple gene-expression values, such as MEAN and VAR, are more abundant than features resulted from operators that only pick one gene (e.g., MIN, MAX, and MEDIAN), as the later can be more affected by noise in the network structure.

### Prediction performance of different feature types

**Fig. 3:**
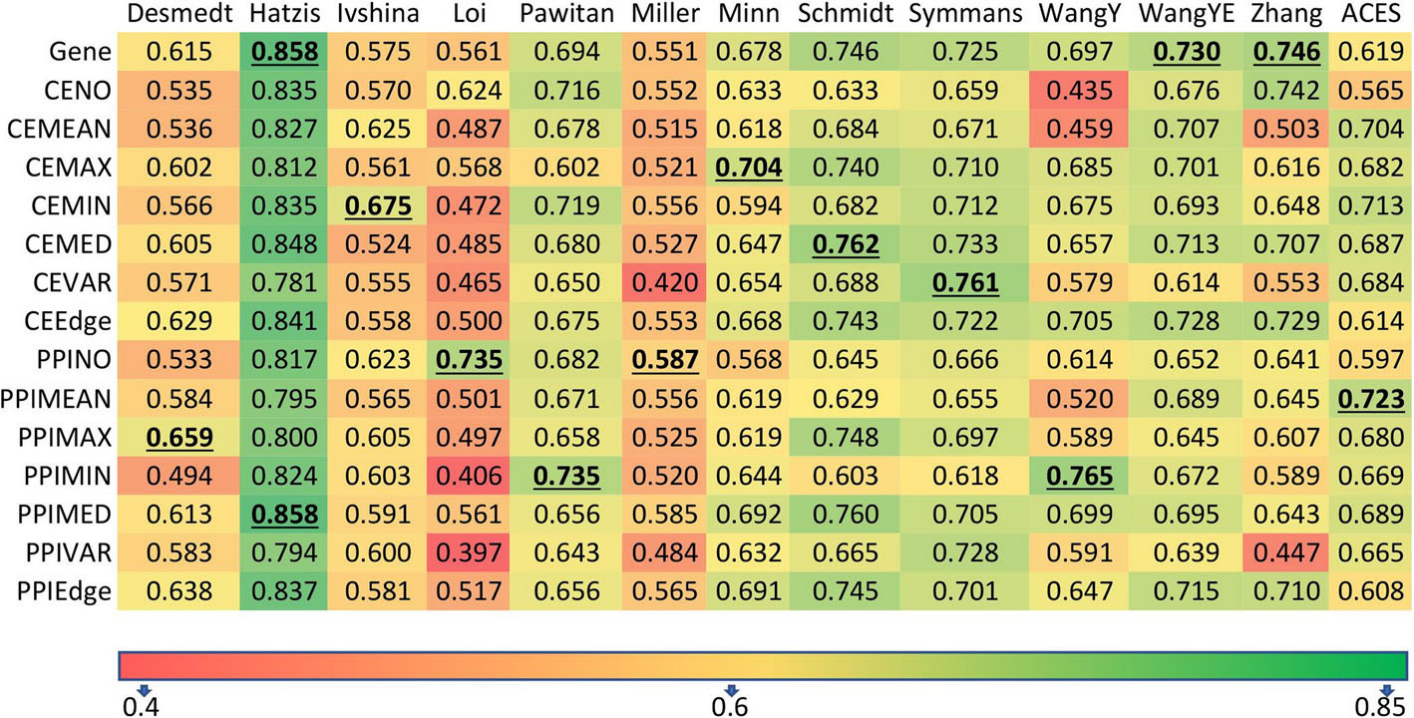
Classification accuracy of each feature type in 13 datasets. The highlighted entry as “bold” and “underlined” in each dataset indicates that it has the highest average AUC score for that dataset

The second analysis was focused on whether the integration of the network with genes offers better classification accuracy compared to gene-based feature (Fig. 3). Out of the 12 independent datasets, gene-based feature and network-based features had the highest AUC scores on 3 and 9 datasets respectively. For example, PPIMAX is the highest in “Desmedt” dataset whereas CEMIN is the highest in “Ivshina” dataset. Different feature types achieved the highest AUC score in different datasets among the network-based feature types which indicated that the classification accuracy varied across the datasets. No feature type showed consistent improvement across datasets. Although network-based features outperformed gene-based feature in 9 datasets, it is insufficient to claim the effectiveness of network-based over gene-based features among the different studies, given that multiple types of network-based features were tested against the gene-based feature.

**Fig. 4:**
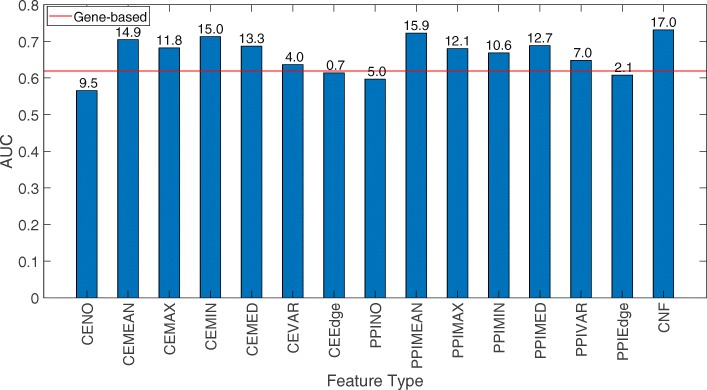
Comparison of classification accuracy of different network-based features with the gene-based feature for ACES dataset. The red line indicates the average AUC score of gene-based feature. The bars indicate the average AUC score of 10 repetitions for 5-fold cross-validation. The value on top of the bar indicates the -log10(*p*-value) of the two-sided paired t-test of the AUC scores of the cross-validation folds of indicated feature type with the gene-based feature

However, when tested on the combined *ACES* dataset, which is relatively free from class imbalance problem and had the highest patient cohort diversity, the network features based on MEAN, MIN, MAX, and MEDIAN operators of gene and its neighbors from both co-expression network and protein-protein interaction network significantly outperformed gene-based feature (Fig. 4). VARIANCE-based features from both PPI and co-expression networks also provided slightly better AUC than the gene-based feature. While CEEdge and PPIEdge features performed on par with the gene-based feature, it is worth noting that the number of features in these feature types is much larger than the other types of features, which could have impeded their classification accuracy. More investigation may be needed to understand the ways of finding a subset of edges that will offer improved classification accuracy. Finally, Neighbor-Only features (CENO, PPINO) do not seem to have any advantage over gene-based features in improving classification performance. This is also consistent with the results that very few Neighbor-Only features are statistically significant between good and poor outcome groups (Fig. 1).

Overall, using *ACES* dataset, we showed that integration with PPI or co-expression networks resulted in not only larger numbers of significant features than using gene expression data alone, but also more accurate classification models. This is consistent with the results reported in [21], but in contradiction with the findings in two previous studies, where network-based features were shown to provide no benefit in classification [23, 24]. The discrepancy may be attributed to the fact that we did not perform any feature selection for any of the feature types in our study in contrast to other studies. Supervised feature selection as performed in other studies may introduce overfitting, given a large number of features and a modest number of samples.

As the MEAN, MIN, MAX, and MEDIAN operators on both gene co-expression and protein-protein interaction networks seem to be beneficial but mutually complementary to each other (data not shown), we tested whether the combination of these features can improve prediction performance further. Indeed, by merging the classifiers from different network-based features into an ensemble classifier (CNF), the highest classification AUC was observed on the *ACES* dataset (Fig. 4), signifying the benefit of combining different network features for improved prediction accuracy.

### Classification accuracy on smaller sub-samples of ACES dataset

**Fig. 5:**
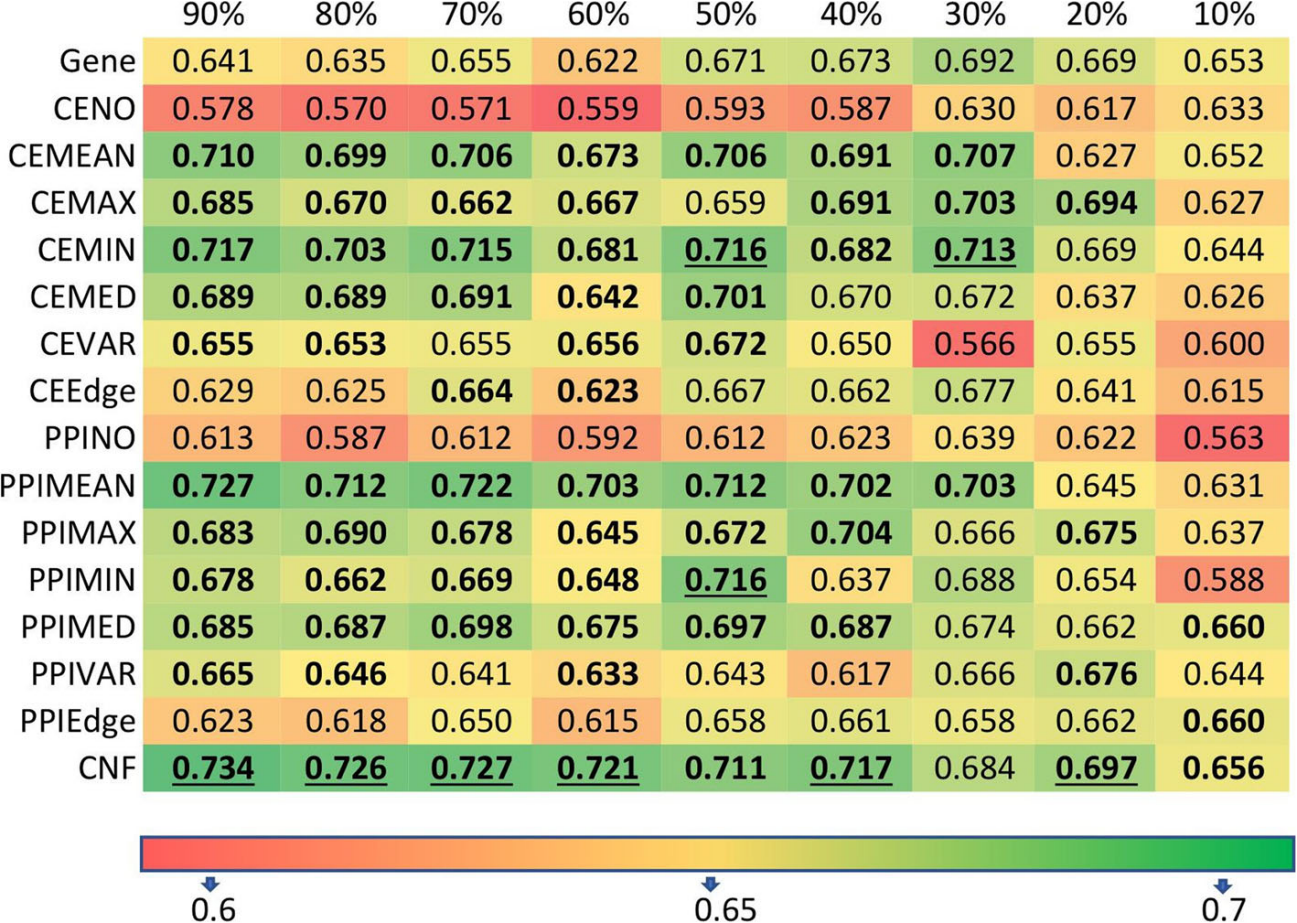
Classification accuracy on different sub-samples of ACES. The highlighted entry as “bold” indicates that the average AUC for that network-based feature is higher than the average AUC score of gene-based feature and the entry as “underlined” indicates that it has the highest AUC score for that sub-sample

To understand why the network-based features worked well in the combined *ACES* dataset, but not in many of individual cohorts, the effect of sample sizes on classification accuracy was investigated to determine its relationship with the classification accuracy of network-based features. For this analysis, 10 sub-samples consisting of the same number of patients were created for specific percentages (from 90% down to 10%) of the *ACES* dataset. Then, similar 5-fold cross-validations were performed for 10 repetitions of each of the 10 sub-samples. The average AUC scores of each feature type and different sub-sample percentages are shown in Fig. 5. From Fig. 5, it can be observed that while gene-based feature have relatively stable performance with regard to different sample sizes, the network-based features, in particular, MEAN, MAX, MIN, and MEDIAN, from both co-expression and PPI networks outperformed gene-based feature only for relatively larger sample sizes, and can be much worse than gene-based feature for small sample sizes. Even more consistent trend is observed on the ensemble classifier combining different network features: average AUC for the CNF classifier decreases steadily with the decrease of sample sizes. Given that no consistent improvement of network-based features over gene-based feature was found on the 12 individual cohorts (Fig. 3), it can be concluded that a minimum number of samples are needed to obtain an improved and stable prediction accuracy using network-based features. This results aligns well with other studies where datasets were merged together for improved accuracy [9, 10].

### Robustness of features across patient cohorts

**Fig. 6:**
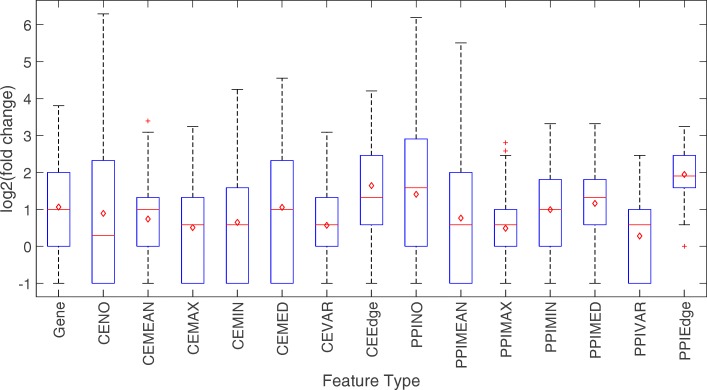
Feature type stability. Boxplot of the fold change of overlapping gene signatures in pairwise setting across 12 studies. Fold change values were converted to log scale. Red diamond denotes the geometric mean of the fold change values

Figure 6 shows the robustness value for different feature types. Overall, network-based features do not seem to have significantly higher overlap than gene-based feature, contrary to claims made by several recent studies [12, 21, 27], but consistent with results reported in [23]. Three network-based features, CEEdge, PPIEdge, and PPINO appear to be the most robust among the 15 feature types and the extent of overlap is higher than gene-based features. However, further investigation revealed that the top-ranked CEEdge and PPIEdge features tend to involve genes with higher degree (mean degree equal to 43 and 102, compared to the network average of 25 and 28, for co-expression network and PPI network, respectively). Therefore, the improved robustness may simply reflect a selection bias as there are few hub nodes in each network, which increases the chance of feature overlap between different datasets. Similarly, the improved robustness of PPINO was due to an inherent bias in including low-degree genes, where the median degree of the top-160 features was zero in 6 out of the 12 studies. We suspect that this type of biases is the main reason for the high robustness of network-based features claimed in the literature and a more comprehensive re-evaluation of the previously reported results may be deserved.

### Gene ontology (GO) analysis of the feature types

To investigate the biological function of the top-ranked features, we combined the top-160 genes for each feature type from all 12 cohorts and performed gene ontology enrichment analysis using the combined gene list. Tables 3 and 4 show top-ranked GO terms along with FDR-corrected *p*-values for gene- and network-based features respectively. Overall, cell cycle and DNA damage are the most recurring GO terms, appearing in most feature types. Other well-known metastasis-related terms such as cell-cell adhesion, cell junction, and various signaling pathways including p53 and Jak-stat appear in several feature types, but the statistical significance of the enrichment is rather low, partly due to the FDR correction required to tackle with the multiple hypothesis testing problem. This is in agreement with the low robustness of the features across datasets, and suggests that a combination of diverse features from large cohorts as in this study is necessary for both mechanistic understanding and improved prediction of metastatic breast cancer.

**Table 3:** GO analysis of gene-based feature

Feature	Term	Count	Benjamini corrected *p*-value
Gene-based	Cell cycle	138	2.20E-15
	DNA repair	42	1.90E-01
	cell-cell adhesion	49	4.4.E-1
	p53 signaling pathway	20	1.70E-02

**Table 4:** GO analysis of network-based feature types

Feature Type	Co-expression Network			PPI network		
	Term	Count	Benjamini corrected *p*-value	Term	Count	Benjamini corrected *p*-value
NO	Growth factor	24	4.10E-02	G-protein coupled receptor signaling pathway	49	9.40E-01
	Jak-stat signaling pathway	28	1.70E-01	Jak-stat signaling pathway	19	4.30E-01
	Cell junction	49	5.90E-01	Cell junction	42	1.80E-01
	Olfactory transduction	13	6.40E-01	Extracellular region	111	6.60E-02
MEAN	RNA transport	33	3.30E-01	Transmembrane	479	1.60E-24
	Lysosome	40	5.00E-01	Extracellular matrix	37	2.30E-03
	Antigen processing and presentation	19	3.60E-01	Bicellular tight junction	14	8.80E-01
	Endothelial cell chemotaxis	5	9.90E-01	Notch signaling pathway	6	9.2E-1
				Leukocyte transendothelial migration	10	9.7E-01
MAXIMUM	Cell cycle	132	2.40E-12	Cell cycle	122	2.70E-10
	Antigen processing and presentation	27	7.70E-03	Positive regulation of canonical Wnt signaling pathway	36	1.60E-05
	DNA repair	45	5.20E-02	T-Cell receptor signaling pathway	51	1.20E-09
	Cardiac epithelial to mesenchymal transition	5	9.9E-01	Cell-cell adherens junction	70	3.50E-05
				Positive regulation of blood vessel endothelial cell migration	7	5.7E-01
MINIMUM	Cell cycle	116	2.00E-06	Cell cycle	136	5.90E-14
	p53 signaling pathway	5	9.00E-01	p53 signaling pathway	19	4.90E-02
	Rho cell motility signaling pathway	7	9.60E-01	DNA repair	41	2.80E-01
MEDIAN	Cell cycle	124	2.80E-09	Sensory transduction	26	9.90E-01
	DNA repair	47	1.70E-02	Telomere	4	9.80E-01
	CSonic Hedgehog (SHH) Receptor Ptc1 Regulates cell cycle	3	9.90E-01	Ribosome	17	9.80E-01
VARIANCE	Cell cycle	138	6.30E-18	Intracellular steroid hormone receptor signaling pathway	7	9.40E-01
	DNA repair	47	8.80E-04	Extracellular space	177	2.10E-01
	Positive regulation of telomere maintainance	12	8.00E-02	Immune response	64	9.50E-01
EDGE	Cell cycle	147	1.80E-22	Cell cycle	167	4.80E-30
	Positive regulation of telomere maintainance	15	1.30E-03	Cell-cell adherens junction	84	5.00E-10
	DNA repair	44	7.20E-03	Positive regulation of epithelial to mesenchymal transition	7	9.5E-01
	Cell-cell adhesion	48	1.30E-01	Regulation of cell motility	6	9.8E-01

## Conclusions

Improved prediction accuracy and signature stability across multiple datasets are very crucial for the prediction and mechanistic understanding of breast cancer metastasis. Here we present a comprehensive analysis of distinct network-based features in comparison to gene-based feature. In general, the number of patients and the ratio between metastatic and non-metastatic patients in the dataset can dramatically impact the number of significant features that can be detected, for both gene-based and network-based features. While network-based features can provide higher prediction accuracy than gene-based features in large cohorts, its performance gain diminishes in smaller dataset. We did not find strong evidence to support the claim that network-based features are more stable than gene-based feature (in fact, some potential bias that could have lead to the false claim was identified). In addition, gene ontology analysis revealed relatively insignificant enrichment of known metastasis-related pathways. Nevertheless, an ensemble classifier combining different network features achieved significantly higher accuracy than gene-based and individual network-based features, signifying both the potential and challenges in network-based prediction and understanding of breast cancer metastasis.

## Data Availability

The datasets used and/or analysed during the current study are available from the corresponding research article.
